# The progesterone receptor Val660→Leu polymorphism and breast cancer risk

**DOI:** 10.1186/bcr928

**Published:** 2004-09-22

**Authors:** Immaculata De Vivo, Susan E Hankinson, Graham A Colditz, David J Hunter

**Affiliations:** 1 Channing Laboratory, Department of Medicine, Brigham and Women's Hospital, Boston, MA, USA; 2 Department of Epidemiology, Harvard School of Public Health, Boston, MA, USA; 3 Program in Molecular and Genetic Epidemiology, Harvard School of Public Health, Boston, MA, USA; 4 Department of Nutrition, Harvard School of Public Health, Boston, MA, USA

**Keywords:** breast cancer, linkage disequilibrium, polymorphism, progesterone receptor

## Abstract

**Background:**

Recent evidence suggests a role for progesterone in breast cancer development and tumorigenesis. Progesterone exerts its effect on target cells by interacting with its receptor; thus, genetic variations, which might cause alterations in the biological function in the progesterone receptor (*PGR*), can potentially contribute to an individual's susceptibility to breast cancer. It has been reported that the PROGINS allele, which is in complete linkage disequilibrium with a missense substitution in exon 4 (G/T, valine→leucine, at codon 660), is associated with a decreased risk for breast cancer.

**Methods:**

Using a nested case-control study design within the Nurses' Health Study cohort, we genotyped 1252 cases and 1660 matched controls with the use of the Taqman assay.

**Results:**

We did not observe any association of breast cancer risk with carrying the G/T (Val660→Leu) polymorphism (odds ratio 1.10, 95% confidence interval 0.93–1.30). In addition, we did not observe an interaction between this allele and menopausal status and family history of breast cancer as reported previously.

**Conclusion:**

Overall, our study does not support an association between the Val660→Leu PROGINS polymorphism and breast cancer risk.

## Introduction

Until recently, the role of progesterone on mammary gland tumorigenesis was not well understood. Data from epidemiological studies revealed a higher risk for breast cancer in postmenopausal women who used a combination of estrogens and progestins, in comparison with those women who used estrogens alone [1,2]. As demonstrated in the progesterone receptor knockout mouse, the physiological effects of progesterone are completely dependent on the presence of its receptor gene, *PGR*, which exists as a single-copy gene. The *PGR *gene uses separate promoters and translational start sites to produce two protein isoforms, hPR-A and hPR-B [3-5], that are identical except for an additional 165 amino acids present only in the amino terminus of hPR-B [6,7]. Although hPR-B shares many important structural domains with hPR-A, the two isoforms are functionally distinct transcription factors [8] that mediate their own response genes and physiological effects with little overlap [9,10]. The progesterone receptor knockout mouse, in which the functional activity of both hPR-A and hPR-B were simultaneously ablated, revealed that progesterone is required for the formation of ductal and alveolar structures during pregnancy [11,12]. Selective ablation of PR-B in a mouse model, resulting in the exclusive production of PR-A, revealed that PR-B is necessary for breast formation [13]. Given the evidence described above for the role of progesterone in breast cancer causation, we proposed that variations in the *PGR *gene might predispose women to breast cancer. Several studies have investigated the Val660 →Leu G/T polymorphism and the PROGINS Alu insertion, which are in complete linkage disequilibrium [14], in association with breast cancer [15-17]. In this study we focused on the Val660→Leu G/T polymorphism that has been reported to be associated with a decreased risk of breast cancer [17].

## Materials and methods

Detailed information about this nested case-control study and exposure data has been reported previously [18]. The protocol was approved by the Committee on Human Subjects, Brigham and Women's Hospital. Genotyping assays were performed by the 5' nuclease assay (TaqMan^®^) by the ABI PRISM 7900HT Sequence Detection System (Applied Biosystems, Foster City, CA). TaqMan primers, probes, and conditions for genotyping assays are available from the authors on request. Genotyping was performed by laboratory personnel blinded to case-control status, and blinded quality control samples were inserted to validate genotyping procedures. Concordance for the blinded samples was 100%.

Odds ratios (ORs) and 95% confidence intervals (CIs) were calculated by using conditional and unconditional logistic regression. In addition to the matching variables, we adjusted for breast cancer risk factors: body mass index (BMI) (kg/m^2^) at age 18 years, weight gain since age 18 years, age at menarche, parity/age at first birth, duration of postmenopausal hormone use, first-degree family history of breast cancer, and history of benign breast disease. We also adjusted for age at menopause in analyses limited to postmenopausal women. Indicator variables for all genotypes were created by using the wild-type genotype as the reference category in the regression models. Because of the low prevalence of homozygous variants, we combined heterozygotes and homozygotes in the logistic regression analysis. Interactions between genotypes and breast cancer risk factors were evaluated by including appropriate interaction terms in unconditional logistic regression models. The nominal likelihood ratio test was used to assess the statistical significance of these interactions. We used SAS version 8.0 (SAS Institute, Cary, NC) for all analyses. We tested Hardy–Weinberg agreement by using a χ^2 ^test.

## Results and discussion

Our study included a total of 1323 incident breast cancer cases, diagnosed after blood draw to 1 June 2000, and 1854 matched controls. Of these, 1134 cases and 1640 controls were postmenopausal at blood draw, and 112 cases and 121 controls were premenopausal; menopausal status was uncertain in 77 cases and 93 controls. The mean age of cases at blood draw was 57.3 years; for controls it was 57.9 years. Cases and controls had similar mean BMI at blood draw (25.5 versus 25.5 kg/m^2^) and weight gain since age 18 years (11.6 versus 11.3 kg). In comparison with controls, cases had similar ages at menarche (12.5 versus 12.6 years), first birth (23.0 versus 23.0 years) and age at menopause (48.2 versus 47.9 years). The proportion of women with a first-degree family history of breast cancer was significantly higher among the cases (20.0% versus 15.0%). Cases were also more likely to have a history of benign breast disease (64.0% versus 51.0%) and a longer duration of postmenopausal hormone use (50.3% versus 49.7% current users for five or more years). The study population was predominantly Caucasian (89% of cases, 86% of controls).

The prevalence of the variant carriers was similar to that in a previous report for Caucasian women [14]: 31% for the cases and 29% for the controls. The genotype distribution of the Val660→Leu polymorphism among the cases and controls was in Hardy–Weinberg equilibrium. We did not observe a statistically significant association of breast cancer among carriers of the Val660→Leu G/T polymorphism. Too few homozygote variants were available in which to analyze the heterozygotes and homozygotes separately. Compared with the G/G wild-type genotype, the adjusted OR for women with G/T and T/T was 1.10 (95% CI 0.93–1.30) (Table 1). Because the previously reported inverse association was confined to premenopausal women, we stratified by menopausal status and observed no association among premenopausal women (adjusted OR 1.21 [95% CI 0.64–2.28]) although we had a relatively small number of women for this analysis (Table 1). The Val660→Leu polymorphism has been suggested to modify the association between family history of breast cancer and breast cancer [17]. We observed no statistically significant interactions between the Val660→Leu polymorphism and first-degree family history of breast cancer. In addition, we selected BMI, history of benign breast disease, and hormone replacement therapy use among postmenopausal women as potential effect modifiers based on biological plausibility. We observed no significant interactions between the Val660→Leu polymorphism and any of these risk factors.

**Table 1:** Association between the progesterone receptor exon 4 (Val660→Leu) G/T polymorphism and breast cancer risk

Genotype^a^	Cases, *n *(%)	Controls, *n *(%)	Crude OR (95% CI)	Adjusted OR (95 % CI)
*G/G *(Val/Val)	869 (69)	1186 (71)	1.0^b^	1.0^c^
*G/T*+*T/T*(Val/Leu+Leu/Leu)	383 (31)	474 (29)	1.08 (0.92–1.27)	1.10 (0.93–1.30)
Premenopausal women				
*G/G*(Val/Val)	68 (65)	79 (72)	1.0^d^	1.0^e^
*G/T*+*T/T*(Val/Leu+Leu/Leu)	36 (35)	31 (28)	1.35 (0.75–2.41)	1.21 (0.64–2.28)
Postmenopausal women				
*G/G*(Val/Val)	745 (69)	1044 (71)	1.0^f^	1.0^g^
*G/T*+*T/T*(Val/Leu+Leu/Leu)	330 (31)	427 (29)	1.09 (0.92–1.29)	1.08 (0.91–1.28)

^a^Numbers may vary because of missing genotypes.

^b^Conditional logistic regression adjusted for the following matching variables: age, menopausal status, postmenopausal hormone use at blood draw, date at blood draw, time at blood draw, and fasting status.

^c^Conditional logistic regression adjusted for matching variables and age at menarche, age at menopause, age at first birth/parity, Body Mass Index (BMI) at age 18 years, weight gain since age 18 years, benign breast disease, first-degree family history of breast cancer, and duration of postmenopausal hormone use.

^d^Unconditional logistic regression adjusted for matching variables listed in footnote b.

^e^Unconditional logistic regression adjusted for matching variables in footnote b and other covariates listed in footnote c.

^f^Unconditional logistic regression adjusted for the following matching variables: age, menopausal status, postmenopausal use at blood draw, date at blood draw, time at blood draw, and fasting status.

^g^Unconditional logistic regression adjusted for matching variables in footnote f and age at menarche, age at first birth/parity, BMI at age 18 years, weight gain since age 18 years, benign breast disease, first-degree family history of breast cancer, and duration of postmenopausal hormone use.

## Conclusions

Our data do not support an inverse association between the Val660→Leu polymorphism and breast cancer risk as reported previously [17]. These results are consistent with recent studies of mostly Caucasian women in which no association was observed between this polymorphism and breast cancer risk in either premenopausal or postmenopausal women [15,16,19,20]. Most notable was the study by Spurdle and colleagues, in which a substantial number of premenopausal cases (*n *= 769) were evaluated [19]. We had limited power to study this association in premenopausal women, but this is the largest study of postmenopausal women reported so far. The large sample size, prospective design and extensive relevant life-style information are among the strengths of this study. In conclusion, our results suggest that there is no association between the Val660→Leu polymorphism and breast cancer risk despite the substantial power of the study (more than 80% power to detect an OR of 0.75 or less for the carrier genotype).

## Competing interests

None declared.

## Abbreviations

BMI = body mass index; CI = confidence interval; LD = linkage disequilibrium; OR = odds ratio PGR = progesterone receptor.
